# Single Cell RNA Sequencing in Autoimmune Inflammatory Rheumatic Diseases: Current Applications, Challenges and a Step Toward Precision Medicine

**DOI:** 10.3389/fmed.2021.822804

**Published:** 2022-01-18

**Authors:** Tadeja Kuret, Snežna Sodin-Šemrl, Brane Leskošek, Polonca Ferk

**Affiliations:** ^1^Faculty of Medicine, Institute of Cell Biology, University of Ljubljana, Ljubljana, Slovenia; ^2^Department of Rheumatology, University Medical Centre Ljubljana, Ljubljana, Slovenia; ^3^Faculty of Mathematics, Natural Sciences and Information Technologies, University of Primorska, Koper, Slovenia; ^4^Faculty of Medicine, Institute for Biostatistics and Medical Informatics/ELIXIR-SI Center, University of Ljubljana, Ljubljana, Slovenia

**Keywords:** single cell, RNA sequencing, rheumatoid arthritis, systemic lupus erythematosus, systemic sclerosis, precision medicine

## Abstract

Single cell RNA sequencing (scRNA-seq) represents a new large scale and high throughput technique allowing analysis of the whole transcriptome at the resolution of an individual cell. It has emerged as an imperative method in life science research, uncovering complex cellular networks and providing indices that will eventually lead to the development of more targeted and personalized therapies. The importance of scRNA-seq has been particularly highlighted through the analysis of complex biological systems, in which cellular heterogeneity is a key aspect, such as the immune system. Autoimmune inflammatory rheumatic diseases represent a group of disorders, associated with a dysregulated immune system and high patient heterogeneity in both pathophysiological and clinical aspects. This complicates the complete understanding of underlying pathological mechanisms, associated with limited therapeutic options available and their long-term inefficiency and even toxicity. There is an unmet need to investigate, in depth, the cellular and molecular mechanisms driving the pathogenesis of rheumatic diseases and drug resistance, identify novel therapeutic targets, as well as make a step forward in using stratified and informed therapeutic decisions, which could now be achieved with the use of single cell approaches. This review summarizes the current use of scRNA-seq in studying different rheumatic diseases, based on recent findings from published *in vitro, in vivo*, and clinical studies, as well as discusses the potential implementation of scRNA-seq in the development of precision medicine in rheumatology.

## Introduction

The beginning of the 21^st^ century was marked by the introduction of new generation sequencing (NGS) technology, leading the way toward a new chapter in genomic and transcriptomic research. NGS technology enabled routine sequencing, quantifying and analyzing millions of transcripts simultaneously in different cell mixtures and tissues (1), while RNA sequencing (RNA-seq) has become a fundamental tool for performing transcriptome-wide analysis of differential transcript expression and mRNA splicing in physiological and pathological states (2). However, with conventional or bulk RNA-seq the average gene expression level for each transcript in a sample is determined, consisting of a large and heterogeneous population of cells (3). These results reflect the gene expression signatures of predominant cell types in the sample, while the transcriptomic information of rare cell subpopulations and cell-to-cell variability are lost (4). This hampers the precise characterization of a tissue composition in health and disease and thus limits our understanding of disease development and pathology, to the majority of cells present in the tissue (5).

To overcome this problem, a revolutionizing new technology allowing analysis of the whole transcriptome at a resolution of an individual cell, was introduced in 2009 (6) and gained widespread popularity in 2014, with the development of microdroplet method and subsequent lower cost and higher throughput (7). Single cell RNA sequencing (scRNA-seq) now represents an indispensable tool to study complex biological systems (e.g., the immune system) at a single cell level, allowing the discovery of rare and novel cell types, simultaneous characterization of multiple different cell states, and more accurate and integrated understanding of their roles in tissue homeostasis (8). The ability of scRNA-seq to determine the gene expression patterns and molecular events within an individual cell, in contrast to a pooled sample of cells, is transforming our understanding of disease pathology, as well as mechanisms of drug resistance that will eventually lead to development of more targeted therapies (9). The enormous power of scRNA-seq technology has been proven especially in biological systems where cellular heterogeneity is a key aspect, such as immunology and autoimmunity, stem cell biology, and tumor cell biology (4).

In this review, we briefly introduce the development of scRNA-seq, and outline its main concepts, workflows, advantages and challenges. We also provide a detailed description of the current applications of scRNA-seq in autoimmune inflammatory rheumatic diseases, including rheumatoid arthritis (RA), systemic lupus erythematosus (SLE), and systemic sclerosis (SSc). Finally, we discuss the potential implementation of scRNA-seq to facilitate the development of precision medicine in rheumatology.

## Overview of SCRNA-SEQ Technology

The first single cell transcriptome analysis based on the NGS platform was described in 2009, when Tang et al. (6) combined high throughput RNA-seq technology with single cell cDNA amplification for studying early developmental stages of a mouse embryo. They discovered that scRNA-seq technology can determine the expression of 75% (5,270) more genes in a mouse blastomere than a microarray and identified 1,753 previously unknown splice junctions (6). Since then, the scRNA-seq technology has developed immensely with substantial improvements in protocols, resolution, throughput and precision (10, 11). Now, we have the possibility to analyse the transcriptome of thousands of single cells simultaneously with greater depth and accuracy. In addition to the transcriptome, other single cell molecular technologies that enable unbiased screening of the genome, DNA methylation, chromatin accessibility, and spatial resolution of gene expression are significantly expanding as well (12).

The rapid development and increased popularity of scRNA-seq methodology also led to a large number of existing protocols and commercially available scRNA-seq platforms, each with their own advantages and limitations. The variety of available scRNA-seq methods makes the selection of the most appropriate platform for a study challenging, despite being one of the crucial steps for achieving desired research results. The selection greatly depends on the research question, biological sample, as well as available funding. Various protocols and scRNA-seq methods/platforms with their sensitivity, throughput and cost have been extensively reviewed elsewhere (13–16). It is beyond the scope of this article to describe the technology in detail, but it is necessary to mention some of its main aspects, workflows, and limitations in order to allow further discussion of its application in studying rheumatological disorders.

### Typical scRNA-seq Workflow

The typical workflow of scRNA-seq consists of the following major steps: isolation of single cells, messenger RNA (mRNA) capture, reverse transcription and cDNA amplification, library preparation, high throughput sequencing, and bioinformatic data analysis. Different scRNA-seq platforms utilize different technologies to capture and physically separate single cells into individual compartments (reaction units), as well as different chemistry to amplify and create libraries for sequencing (13, 17, 18).

#### Single Cell Isolation

The isolation of viable and intact single cells from the tissue of interest is a crucial and limiting step, often achieved by enzymatic treatment and/or mechanical agitation (13). Previously, scRNA-seq analysis was thought to require cells, isolated from fresh tissue samples, which substantially limited the human tissue collection. Recently, however, standardized cryopreservation protocols ensuring preserved integrity of the specimen were implemented. This is especially beneficial for performing a larger, multi-center study across multiple physically distant institutions in order to obtain a higher number of cells and samples for scRNA-seq. For example, a standardized cryopreservation protocol for skin samples was established by Mirizio et al. (19). The authors compared transcriptomes of cells obtained from skin tissues, preserved either in CryoStor® CS10 cell preservation medium (frozen) or placed in Roswell Park Memorial Institute (RPMI) medium (fresh). The cryopreserved skin cells had comparable cell viability and yield to freshly prepared single cell solutions. More importantly, gene expression signatures were correlated and conserved across all 18 identified cell clusters. However, cryopreservation negatively affected the keratinocyte populations (19). The quality of preservation observed in the skin samples is in line with that observed by Donolin et al. (20) for synovial tissues. The CryoStor® CS10 preserved synovial tissue-derived cells retained intact transcriptomes and cell surface phenotypes (20). CryoStor® CS10 preservation offers an acceptable alternative to fresh tissue for single cell isolation and sequencing,

Once a suspension of single cells is obtained, there are several methods that can be used to deliver each cell into an individual reaction compartment or unit. The traditional methods include limiting dilution, micromanipulation, laser capture microdissection (LCM), and flow-activated cell sorting (FACS) (21). Although the traditional single cell isolation methods can be used to separate individual cells into compartments, the downstream analytical processes, such as cell lysis, reverse transcription, and library construction cannot be performed directly in these compartments, requiring an extra step or equipment, which is prone to introducing errors and may lead to significant material loss (4, 11).

**Figure 1:**
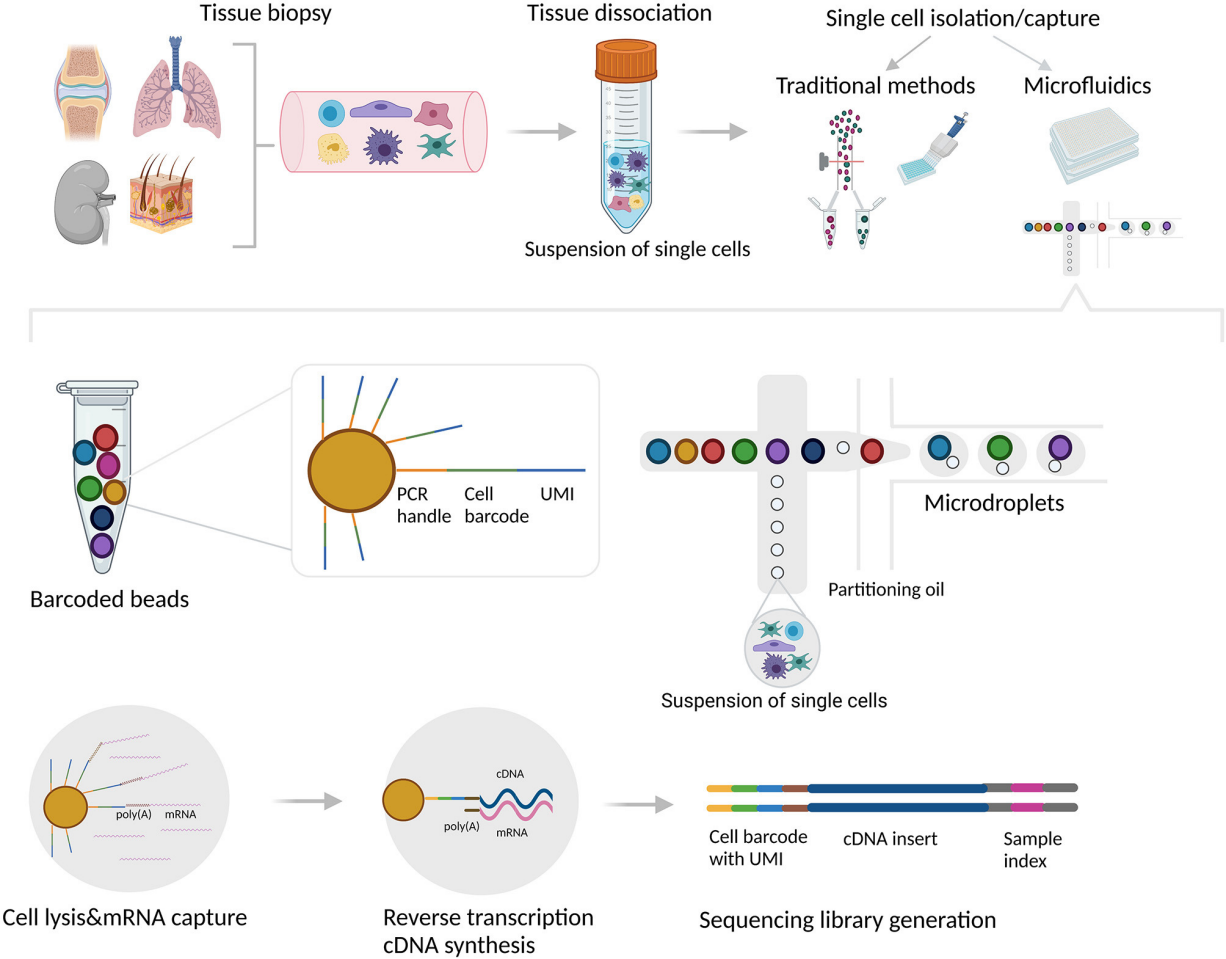
Single cell isolation, capture and library generation using droplet-based microfluidic system. Tissue biopsies are usually dissociated mechanically and/or enzymatically. Single cells can be subsequently isolated and captured into individual reaction units using various methods/platforms. The traditional methods include limiting dilution, micromanipulation, laser capture microdissection (LCM), and flow-activated cell sorting (FACS). Although these methods can be used to separate individual cells into compartments, the downstream analytical processes (cell lysis, reverse transcription, and library construction) cannot be performed directly in these compartments. In contrast, microdroplet technology can be used for both: to capture each individual cell into one compartment/reaction unit, as well as to perform downstream reactions directly in every unit. In the droplet-based microfluidic system (i.e., 10x Genomics Chromium), aqueous droplets are formed in a continuous oil phase. Each droplet contains individual cell mixed with gel beads containing oligo sequences for a bead-specific barcode, unique molecular identifier (UMI) and poly-dT sequence which hybridizes with poly(A) tails of each mRNA. With reverse transcription, the bead-specific barcode integrates into the cDNA, allowing subsequent identification of the cell origin. The figure was created with BioRender.com.

Microfluidic devices, on the other hand, can be used for both: to capture each individual cell into one compartment/reaction unit, either a microwell or a microdroplet, as well as to perform downstream, highly standardized and automated reactions directly in every unit (22, 23). Microdroplet technology (i.e., 10x Genomics Chromium) encapsulates each aqueous droplet in a continuous oil phase which contains an individual cell mixed with gel beads with uniquely barcoded set of oligonucleotides, called unique molecular identifiers (UMIs). The mRNAs from the lysed cell bind to the bead oligonucleotides, and with reverse transcription, the bead-specific barcode integrates into the cDNA, allowing subsequent identification of the cell origin after pooling (24–26). Microwell-based technology (i.e., the BD Rhapsody system and Fluidigm C1) has a similar concept with microwells instead of microdroplets. Single cells in suspension are captured into microwell arrays that contain barcoded magnetic beads, comparable to the gel beads used in microdroplet technology (27). Microfluidic technology has gained widespread popularity due to the low sample volume required and low analysis cost (5), however its major limitations are the introduction of empty compartments, and/or the inclusion of two (doublet) or more cells in one compartment (resulting in two different transcriptome profiles assigned to a single cell), leading to systematic error in the scRNA-seq analysis (28). Single cell isolation and the droplet-based microfluidic platform are illustrated in Figure 1.

#### Reverse Transcription and cDNA Amplification

Isolated individual cells are subsequently lysed to release as many intact RNA molecules as possible, followed by mRNA enrichment, usually by oligodT priming. The mRNAs originating from the same cell are tagged with the same UMIs in order to differentiate between transcripts from different cells (29) (Figure 1). Reverse transcriptase with low RNase H activity and increased thermostability is frequently used for reverse transcription of mRNA and first strand cDNA synthesis, while the second strand can be generated using different strategies (5). One approach includes a template-switching mechanism at the 5' end of the RNA template (e.g., SMART technology) (29, 30), while another strategy uses either poly(A) or poly(C) tails to ligate the 5' end of cDNA and generate common adaptors for downstream PCR amplification (6, 31).

PCR is typically used to amplify cDNA from small amounts of input material. However, the exponential amplification can potentially bias the representation of gene expression profiles toward shorter amplicons with a lower amount of G-C paired bases (13, 32). To avoid this, UMIs have been introduced. Using UMIs, each transcript can be assigned to its cell of origin, which eliminates PCR bias and improves accuracy (26, 29, 33). As an alternative, *in vitro* transcription that uses T7 RNA polymerase and ensures linear amplification can be performed instead of PCR, but it is time consuming as it requires an additional reverse transcription and may lead to 3′ coverage biases (5, 34, 35).

#### Library Preparation and High Throughput Sequencing

Amplified and tagged cDNAs from every cell are pooled and sequenced by NGS, using library preparation techniques and sequencing platforms similar to those used for bulk RNA-seq. In general, the methods for cDNA sequencing library preparation can be categorized into two groups: i) full-length and ii) molecular tag-based (13). Full-length methods (e.g., SMART-seq, SMART-seq2, SUPeR-seq, and MATQ-seq) cover the entire transcriptome and are thus suitable for cell-type and isoform discovery, as well as allelic gene expression analysis. However, by using the full-length sequencing method, the samples cannot be combined with UMIs, multiplexed and pooled into a single tube for library generation, thus increasing overall cost and labor intensity (17, 33, 36). On the other hand, molecular tag-based methods (e.g., MARS-seq, STRT-seq, CEL-seq, CEL-seq2, Drop-seq, inDrops) can be combined with UMIs, since they sequence either the 5′ or 3′ end of the transcript and thus enable multiplexing and sample pooling, and consequently reduce cost as well as allow high throughput (17, 25, 37).

#### Bioinformatic Data Analysis

Although scRNA-seq is now becoming more accessible to many laboratories through commercially available reagents and platforms, computational protocols are still very limited and data analysis requires experienced personnel that can adapt fast with appropriate bioinformatics tools and pipelines required for specific scRNA-seq usage (38). ScRNA-seq data processing usually includes two main procedures: i) pre-processing, including quality control of raw reads, batch effect correction, normalization, read alignment, and gene expression quantification, and ii) downstream analysis consisting of differential expression and gene set enrichment analysis, subpopulation clustering, cell cycle phase assignment, reconstruction of cell trajectory and pseudo time (12, 39–41). Once reads are obtained from a sequencing platform, several quality control steps are necessary before further analyses (14). For example, inviable cells and doublets can contaminate the raw data and therefore need to be identified and removed. Batch effect must be corrected as it can introduce systematic error and confound the technical and biological variability, leading to significant differences in gene expression profile and misinterpretation of the data. The next essential step is normalization of the data, to adjust for several factors, such as sequencing depth, gene length, dropouts, and other technical effects (5, 11, 40). After the trimming, mapping the reads to a reference genome, and gene expression quantification, subsequent analyses of feature selection, dimensionality reduction, and visualization can be performed using several different bioinformatic tools, extensively reviewed in (41) and listed in the publicly available database (https://www.scrna-tools.org/), such as Scanpy, Seurat, Cell Ranger, t-SNE, UMAP, and SPRING. Single cells can be either clustered into subpopulations, based on their shared transcriptional signature, or trajectory analysis with pseudo-temporal ordering can be performed to phenotypically identify cellular states (5, 11, 40). A scRNA-seq bioinformatics platform that will cover complete bioinformatic pipeline from raw data generation, and storage, as well as virtualized bioinformatic data analysis with state-of-the-art tools on Linux and R is currently being established as part of the ELIXIR research infrastructure for life science information (https://elixir-europe.org).

### Challenges and Limitations

The first and most challenging step in scRNA-seq is obtaining a high yield of high quality and viable single cells without causing significant alteration in their transcriptional profile. To achieve this, several optimization steps might be required, depending on the type of tissue in question. High cell-to-cell variability is a common problem in scRNA-seq data due to the technical (e.g., RNA capture efficiency, limited amount of RNA present in single cells) and biological noise (e.g., stochastic gene expression, a variety of cellular states, cell sizes and cell cycle phases). We can overcome some of these issues by increasing the number of sequenced single cells and by subgrouping cell populations into clusters. Once the clusters are formed, the data from each cluster can be pooled to give a more sensitive and complete representation of the gene expression pattern (13). Furthermore, batch effects and systematic errors commonly occur in scRNA-seq. Batch effects can be a consequence of sample handling (i.e., cells being sequenced separately at different sequencing depth), using different lots of reagents and several biological specimens. They can be corrected by different computational tools, such as ComBat (42), but should preferably be avoided by adequate experimental design, multiple biological replicates (43, 44) or by pooling cells across experimental conditions and samples with subsequent demultiplexing using cell tagging strategy (45), or via genetic variation (46). Other limitations mostly include high cost of the method, challenges related to bioinformatic data analysis, difficulties in the interpretation of the results and their meaningful translation into a clinical setting. The balance between capturing sufficient amounts of RNA and at the same time, obtaining the fidelity of information, is critical when using scRNA-seq technologies. The results should therefore be carefully analyzed and interpreted, also having in mind various limitations of the technology.

## Application of SCRNA-SEQ in Autoimmune Inflammatory Rheumatic Diseases

Autoimmune rheumatic diseases are chronic, debilitating, and painful conditions associated with considerable morbidity and mortality that affect more than five million individuals worldwide (47). They comprise more than 150 different disorders and predominantly affect the connective tissue, joints, skin and the musculoskeletal apparatus (48). The majority of rheumatic disorders develop as a consequence of an abnormal systemic immune response leading to immune cell activation and differentiation, instructed also by the affected tissue microenvironment (e.g., tissue resident stromal cells) which initiates and perpetuates the inflammatory reaction (49, 50). Considerable heterogeneity within and between the affected tissues complicates the understanding of the underlying mechanisms of disease development, and more importantly, contributes to treatment resistance and failure (4, 51). Therefore, using scRNA-seq to uncover the functional status and exact molecular phenotype of individual cells will be paramount in identifying novel therapeutic targets and specific predictors of treatment responses, leading to a more informed and stratified treatment decision-making process. To successfully implement scRNA-seq and other single cell studies in RA and SLE, the NIH Accelerating Medicines Partnership (AMP) RA/SLE Network was established. The AMP RA/SLE network has already made several important discoveries, as well as protocol optimizations and standardizations, that will facilitate future inter-institutional collaborations (20, 51, 52).

### Rheumatoid Arthritis

Rheumatoid arthritis (RA) is a chronic disease that predominantly affects the joints with recurrent and persistent inflammation that can, if left untreated, eventually lead to joint destruction and disability. Various cell types in the joint are importantly implicated in RA-associated inflammation and tissue destruction, which include synovial fibroblasts, macrophages, lymphocytes, osteoclasts, and vascular endothelial cells. Synovial fibroblasts and macrophages have emerged as the key cells mediating local inflammatory response and destruction in the affected joints and they are currently considered as important therapeutic targets (4, 51, 53, 54).

#### Synovial Tissue-Derived Fibroblasts

The first study, to our knowledge, using scRNA-seq to decipher the heterogeneity of synovial tissue-derived cells in RA joint was published in 2018 by Stephenson et al. (55) (Table 1). By sequencing 20,387 single cells, isolated from joints of five RA patients, they identified two major fibroblast subpopulations (DAF^+^ and THY1^+^), and determined their anatomical positions in the synovial tissue. DAF^+^ fibroblasts were predominantly located in the synovial lining, while THY^+^ fibroblasts populated the sublining region. Distinct anatomical location also indicated different functions, since DAF^+^ fibroblasts had upregulated expression of several genes, particularly important for endothelial cell proliferation and regulation of reactive oxygen species responses, while THY^+^ fibroblasts were enriched in metallopeptidase activity and the organization of the extracellular matrix (55). These findings were confirmed by Mizoguchi et al. (56) and Zhang et al. (52) who discovered that the sublining THY1^+^ fibroblast subset was significantly enriched in patients with RA compared to patients with osteoarthritis (OA). These fibroblasts abundantly secreted pro-inflammatory cytokines, had proliferative and invasive properties, as well as reflected RA disease activity, and correlated with immune cell infiltration in the synovium (56). The exploration of heterogeneity of synovial fibroblasts in RA continued further and it was generally confirmed that RA progression might be driven by the two types of fibroblasts. The sublining THY1^+^ fibroblasts promote severe and persistent inflammatory arthritis, while THY1^−^ fibroblasts, restricted to the synovial lining layer, mediate bone and cartilage damage with little effect on inflammation. It was also shown that deleting or removing both subpopulations of fibroblasts suppressed inflammation and bone erosion in murine models of arthritis (57). A subsequent study showed that the lining and sublining fibroblasts do not separate entirely into two clusters, but are instead overlapped, along a gradient corresponding to their location. The expression of surface fibroblast markers changes based on their proximity to endothelial cells, e.g., the expression of CD90:PRG4 decreases gradually with greater distance from endothelial cells, and this is regulated by endothelium-derived NOTCH signaling. Furthermore, genetic deletion of NOTCH3 or blocking NOTCH3 signaling with a monoclonal antibody inhibited inflammation and prevented joint damage in mice models, proposing NOTCH3 signaling as an important therapeutic target (58).

**Table 1:** Application of scRNA-seq technology in rheumatoid arthritis.

**Sample type**	**Subjects or mouse model (number)**	**Cells sequenced (number)**	**Single cell platform**	**Main findings**	**Reference**
Human synovial tissue	RA patients (*n* = 5)	Synovial cells (*n* = 20,387)	Drop-seq	13 hematopoietic and fibroblast populations, CD55^+^ synovial lining fibroblast and THY1^+^ sublining fibroblasts.	Stephenson et al. (55)
Human synovial tissue	RA patients (*n* = 2), OA patients (*n* = 2)	FACS sorted fibroblasts (*n* = 384)	Smart-seq2	Expanded PDPN^+^CD34^−^THY1^+^ sublining fibroblast subset in RA vs. OA, secrete pro-inflammatory cytokines, are proliferative, and invasive, reflect RA disease activity, and correlate with immune cell infiltration.	Mizoguchi et al. (56)
Mice synovial tissue	STIA mice (*n* = 3)	FACS sorted fibroblasts (*n* = 2,814)	10X Genomics Chromium	Sublining FAPα^+^THY1^+^ fibroblasts drive severe and persistent inflammation, lining FAPα^+^THY1^−^ fibroblasts mediate bone and cartilage damage.	Croft et al. (57)
Human synovial tissue and organoids	RA patients (*n* = 6) OA patients (*n* = 6)	Stromal cells (*n* = 35,153), synovial organoid cells (*n* = 6,412)	10X Genomics Chromium	Fibroblasts display positional identity, regulated by endothelium-derived Notch signaling, blocking Notch3 and/or Notch signaling prevents joint damage.	Wei et al. (58)
Human synovial tissue	RA patients (*n* = 36), OA patients (*n* = 15)	Synovial cells (*n* = 5,262)	CEL-Seq2	Expanded sublining CD34^−^THY1^+^fibroblasts, IL1B^+^ pro-inflammatory monocytes, ITGAX^+^TBX21^+^ B cells and PDCD1^+^ Tph and Tfh cells in RA vs OA.	Zhang et al. (52)
Human synovial tissue	RA patients (*n* = 10), OA patients (*n* = 2)	FACS sorted CD14^+^ cells (*n* = 940)	CEL-Seq2	HBEGF^+^ inflammatory macrophages are the dominant CD14^+^ subset in RA, promote fibroblast invasiveness and contribute to fibroblast-mediated joint destruction.	Kuo et al. (59)
Human synovial tissue	Active RA (*n* = 5), treatment-refractory RA (*n* = 6), remission RA (*n* = 6), UPA (*n* = 4), healthy ctrl (*n* = 4)	FACS sorted synovial macrophages (*n* = 32,000)	10X Genomics Chromium	MerTK^pos^TREM2^high^ and MerTK^pos^LYVE1^pos^ macrophages had gene expression signature enriched in negative regulators of inflammation, abundantly produced inflammation-resolving lipid mediators and induced the repair response of synovial fibroblasts *in vitro*.	Alivernini et al. (60)
Mice synovial tissue	K/BxN serum-induced arthritis mice (ND)	FACS sorted CD45^+^CD11b^+^Ly6G^−^ macrophages (*n* = 7,362)	10X Genomics Chromium	CX3CR1+ lining macrophages display features common to epithelial cells, form an internal immunological barrier, limit the inflammatory reaction and protect the joint.	Culeman et al. (61)
Mice synovial and lung tissue	K/BxN serum-induced arthritis mice (*n* = 15); ovalbumin-induced asthmatic mice (*n* = 8)	FASC sorted CD45^+^ CD11b^+^ Siglec-F^+^ granulocytes (ND)	10X Genomics Chromium	Induction of asthma can cause resolution of arthritis following the occurrence of a specific subset of rEos in the joints, with proresolving features. They are found in blood and synovium of RA patients in remission.	Andreev et al. (62)
Human peripheral blood	RA patients (*n* = 6)	FACS sorted CD19^+^ B cells (*n* = 2,349)	Modified STRT-Seq	ACPA B cells displayed more somatic hypermutations, and upregulated genes promoting class-switching and T cell-dependent response, RF B cells upregulated genes stimulating memory reactivation through innate immune pathways.	Lu et al. (63)
Human peripheral blood	Index RA patient (*n* = 1)	Magnetic bead separated CD8^+^ T cells (ND)	10X Genomics Chromium	A stable mutation in the clonally expanded CD8^+^ T cells, characterized by upregulated expression of cytotoxic gene products and molecules associated with pro-inflammatory signaling in a patient with ACPA-negative destructive RA.	Kelkka et al. (64)
Mice synovial tissue and peripheral blood	antigen-induced arthritis mice (*n* = 1)	Synovial cells (*n* = 8,426), blood cells (*n* = 4,310)	Seq-Well	Shared pathways and upstream regulators (TNF and IFNy) between mice and human synovial cells, no significant overlaps in transcriptional signatures between mice synovial tissue and peripheral blood.	Lee et al. (65)

*ACPA, anti-citrullinated antibodies; ctrl, control; FACS, flow-activated cell sorting; IL, interleukin; IFN, interferon; ND, not defined; OA, osteoarthritis; RA, rheumatoid arthritis; rEos, regulatory eosinophils; RF, rheumatoid factor; STIA, serum transfer-induced arthritis; UPA, undifferentiated arthritis; TNF, tumor necrosis factor; Tfh, T folicular helper cells; Tph, T peripheral helper cells*.

#### Synovial Tissue-Derived Macrophages

A specific macrophage subset, defined as HBEGF^+^ inflammatory macrophages is the dominant CD14^+^ subset found in RA synovial tissues. These macrophages produce inflammatory cytokines, such as IL1β and growth factors, such as heparin binding epidermal growth factor (HBEGF) and epiregulin, that are formed under the influence of resident fibroblasts and the tumor necrosis factor α (TNFα). They also promote fibroblast invasiveness and thus contribute to fibroblast-mediated joint destruction. Moreover, *ex vivo* experiments showed that non-steroidal anti-inflammatory drugs (NSAIDs), like naproxen, inefficiently block TNFα-induced responses of HBEGF^+^ macrophages, suggesting that NSAID therapy promotes a classic pro-inflammatory macrophage phenotype, perpetuating inflammation instead of resolution (59). Alivernini et al. (60) later discovered that there are distinct subsets of synovial macrophages that regulate inflammation and remission in RA. By profiling 32,000 synovial macrophages, they identified four distinct macrophage subpopulations with gene expression patterns that differ between patients with early/active RA, treatment-refractory/active RA, and RA in sustained remission. Two macrophage subpopulations (MerTK^pos^) had gene expression signatures enriched in negative regulators of inflammation. These macrophages abundantly produced inflammation-resolving lipid mediators and induced the tissue repair mechanism in synovial fibroblasts *in vitro*. RA patients in remission with a low percentage of MerTK^pos^ macrophages had an increased risk of disease relapse, indicating that MerTK^pos^ subpopulations could represent a potential treatment strategy for RA (60). In another study, a specific subset of mice synovial macrophages (CX3CR1^+^), different from monocyte-derived macrophages, was found to display characteristics more common to epithelial cells and formed an internal immunological barrier at the synovial lining. CX3CR1^+^ lining macrophages limited the inflammatory reaction in the joint by generating a tight-junction-mediated physical and functional barrier that could protect the joint from destruction (61).

#### Other Immune Cell Types

Focusing on the resolution of inflammation in RA, Andreev et al. (62) discovered that induction of asthma can cause resolution of arthritis following the occurrence of a specific subset of regulatory eosinophils (rEos) in the joints of arthritis-induced mice. These cells have proresolving features and proliferate upon exposure to IL5. RA patients in remission were reported to have rEos in the blood and synovium, while treatment with mepolizumab (anti-IL5 antibody) caused arthritis relapse in RA patients with concomitant asthma (62).

RA is also characterized by production of autoantibodies, such as anti-citrullinated antibodies (ACPA) and rheumatoid factor (RF) by autoreactive B cells. Recently, a published analysis of the B cell frequencies discovered that RF and ACPA B cells are rare in RA blood, but undetectable in healthy controls. ACPA B cells displayed more somatic hypermutations than RF B cells, accompanied by the upregulation of genes that promote class-switching and T cell-dependent responses. On the other hand, RF B cells expressed transcriptional signatures that stimulate rapid memory reactivation through various innate immune pathways. ACPA and RF B cell-enriched transcripts belong to distinct regulatory pathways, indicating that different molecular mechanisms drive ACPA and RF production in RA (63). A subset of RA patients may develop an especially aggressive ACPA-negative destructive RA phenotype that mostly affects larger joints (66). To find biological markers to differentiate this rare type of RA from ACPA-positive, and other subgroups of ACPA-negative RA, whole-exome sequencing and subsequent scRNA-seq were performed to study somatic mutations in CD8^+^ T cells of an index RA patient. A stable mutation in the clonally expanded CD8^+^ T cells was discovered and these cells were characterized by upregulated expression of cytotoxic gene products and molecules associated with pro-inflammatory signaling, suggesting that this particular clone might be important for promoting chronic inflammation. Patients with this type of RA might be more effectively treated with therapeutic approaches that target CD8^+^ T cell-mediated signaling (64).

#### A Dichotomy Between Local and Systemic Inflammatory Signature

Several novel biomarkers for diagnosis and predicting treatment response in RA have been identified by studies using unbiased high throughput approaches, however, translation into a clinical setting has proven to be challenging. This might be associated with differential inflammatory responses observed systemically in peripheral blood and locally in the synovial tissue of arthritic joints, as recently shown by Lee et al. (65). ScRNA-seq data from matched blood- and synovial tissue-derived cells from a mouse model of arthritis revealed that the inflammatory response in peripheral blood does not reflect the local inflammatory reaction in arthritic joints. Since this kind of dichotomy exists between gene expression signatures and pathways in synovial tissue as compared to peripheral blood, the identification of reliable novel biomarkers in RA may require simultaneous analyses of peripheral blood and disease-associated tissue, as well as standardized pipelines and protocols to generate relevant multidimensional data using scRNA-seq (65) (Table 1). A step forward has been made by Donlin et al. (20), who established a robust protocol to obtain a high yield of viable synovial cells, derived from cryopreserved synovial tissues with intact transcriptomes and cell surface phenotypes, which has been adopted and utilized by the AMP RA network (20).

### Systemic Lupus Erythematosus

Systemic lupus erythematosus (SLE) is a prototypical chronic autoimmune disease that can affect multiple organs, including the skin, joints, lungs, and kidneys. The most common and serious manifestation of SLE is lupus nephritis (LN), that affects roughly 50% of patients with SLE and, in 10% of these patients, progresses to end-stage renal disease (67). Due to the highly diverse clinical manifestations (characterized by flares and remission) and unpredictable disease course, clinical management of SLE remains challenging, calling for further multidimensional studies to improve treatment and prognostic decisions (68, 69).

#### Renal Tissue-Derived Cells

The first study utilizing scRNA-seq in SLE was published in 2017 (70), and confirmed the upregulated interferon (IFN) response in renal tubular cells of SLE patients, as well as correlation between IFN-response scores and chronicity index, IgG deposition, and proteinuria. SLE patients who responded to therapy had significantly lower IFN scores compared to nonresponders. Interestingly, the gene expression profiles from keratinocytes, isolated from nonlesional skin of patients with LN, also revealed upregulated IFN response and IFN-inducible genes compared to healthy controls. Skin tissue, which is more accessible compared to a renal biopsy, might therefore be exploited to reveal renal injury and damage (70) (Table 2). A subsequent study from the same group confirmed these findings using a higher throughput method (the Fluidigm 800-well platform instead of 96-well), with increased cell capture, also allowing for the identification of mesangial cell profiles from patients with LN. A high IFN response score and gene signature associated with fibrosis in tubular cells were associated with treatment failure. scRNA-seq of renal tissues determined molecular signatures clinically relevant to prognosis, which could be used to stratify patients into subgroups to provide a more personalized therapeutic procedure according to the molecular phenotype of the patients (71). The IFN signature was further explored by Arazi et al. (72) who found a clear IFN response in most infiltrating leukocytes from renal biopsies that correlated with the IFN signature found in peripheral blood. The study also revealed upregulated expression of chemokine receptors CXCR4 and CX3CR1 in renal tissues of patients with LN, suggesting their potential as therapeutic targets. Gene expression signatures of leukocytes found in urine and renal biopsies significantly correlated, implying that less invasively obtained urine samples might be used instead of renal biopsies (72). A subsequent study confirmed that proteins found in urine can predict the cell composition of the renal immune infiltrate in LN patients after integrating the urine proteomics with the scRNA-seq of renal biopsies. The urine chemokine gradient significantly correlated with the number of renal-infiltrating CD8^+^ T cells. The authors concluded that patient-specific pathways could be noninvasively determined in the urine samples, potentially enabling personalized treatment (73).

**Table 2:** Application of scRNA-seq technology in systemic lupus erythematosus.

**Sample type**	**Subjects or mouse model (number)**	**Cells sequenced (number)**	**Single cell platform**	**Main findings**	**Reference**
Human renal and skin tissue, peripheral blood	SLE patients with LN (*n* = 24), healthy controls (*n* = 5)	Renal cells (*n* = 361), skin keratinocytes (*n* = 329), CD4^+^CD14^+^ PBMC (*n* = 209)	Fluidigm C1 (96-well)	Upregulated IFN response score in renal tubular cells correlated with chronicity index, IgG deposition, and proteinuria, upregulated IFN-response and ISGs also in keratinocytes from skin of LN patients.	Der et al. (70)
Human renal and skin tissue	SLE patients with LN (*n* = 21), healthy controls (*n* = 3)	Tubular cells (*n* = 1,221), skin keratinocytes (*n* = 1,939)	Fluidigm C1/SMART-Seq (800-well)	A high IFN response score and fibrotic signature in tubular cells were associated with treatment failure.	Der et al. (71)
Human renal tissue and urine	SLE patients with LN (*n* = 24), healthy controls (*n* = 10)	FACS sorted CD45^+^ renal leukocytes (*n* = 2,736), CD45^−^CD10^+^ epithelial cells (*n* = 145), CD45^+^ urine leukocytes (*n* = 577)	CEL-Seq2	21 leukocyte subsets found in renal biopsies, IFN signature found in most leukocytes, correlated with that in peripheral blood, upregulated expression of chemokine receptors CXCR4 and CX3CR1 in renal tissues of patients with LN, correlation between gene expression signatures of leukocytes from urine and kidneys.	Arazi et al. (72)
Human renal tissue	SLE patients with LN (*n* = 6)	FACS sorted CD45^+^ renal leukocytes, CD45^−^CD10^+^ epithelial cells (ND)	CEL-Seq2	Proteins found in urine can predict the cell composition of the renal immune infiltrate, the urine chemokine gradient significantly correlated with the number of kidney-infiltrating CD8^+^ cells.	Fava et al. (73)
Human peripheral blood	SLE children (*n* = 33), healthy children (*n* = 11), adult SLE patients (*n* = 8), adult healthy controls (*n* = 6)	PBMCs from children (*n* = 276,000), PBMCs from adults (82,000)	10X Genomics Chromium	Increased expression of ISGs in children with SLE vs healthy controls, ISG^hi^ derived mostly from 8/20 PBMC subpopulations (especially plasma cells), ISG enriched subpopulations were associated with high disease activity in children and adult SLE patients.	Nehar-Belaid et al. (74)
Human peripheral blood	Healthy blood donors (*n* = 2)	Isolated pDCs (*n* = 1,413), stimulated with RNA-IC, IL-3, and IFN-α2b	ddSEQ (Biorad)	RNA-ICs induced type III IFN (e.g., IFN-λ1–3) production in pDCs in a TLR-MyD88-dependent manner, type III IFNs, dominated by IFN-λ1, were exclusively expressed in a specific minor cluster of pDCs, within a subset of the type I IFN expressing pDC, also enriched in genes coding for CCL4, CCL3, TNF, CCL3L3, and IL12A.	Hjorton et al. (75)
Human peripheral blood, mouse spleen	Healthy blood donors (ND), wild type mice (ND)	Human whole blood cells (*n* = 19,266), mice spleen cells (*n* = 18,520), stimulated with IFN-λ2 or IFN-α	10X Genomics Chromium	IFN-λ receptor deletion resulted in significantly lower immune cell activation, and reduced damage of skin and kidneys in lupus mice, only mice neutrophils and human B cells upregulated ISGs in response to stimulation with IFN-λ, IFN-λ activated keratinocytes and mesangial cells to produce chemokines.	Goel et al. (76)
Human peripheral blood	SLE patients (*n* = 3), healthy controls (*n* = 2)	Isolated B cells (*n* = 15,039)	10X Genomics Chromium	IFN signature determined in a subset of switched memory B cells, increased expression of ISGs in multiple B cell clusters from SLE patients, upregulated expression of CD52 in B cells.	Bhamidipati et al. (77)
Human peripheral blood	SLE patients (*n* = 3)	Isolated PBMCs (*n* = 26,925)	10X Genomics Chromium	LDGs significantly drive the type I IFN signature in SLE patients, two subpopulations of LDGs identified in SLE: immature and intermediate mature LDGs, the latter are associated with SLE organ damage and the presence and severity of coronary artery disease.	Mistry et al. (78)
Human peripheral blood	SLE patients (*n* = 2), healthy controls (*n* = 1)	Isolated PBMCs (*n* = 26,925 together with deposited databases)	10X Genomics Chromium	LDGs exhibited the highest ISG activity in SLE PBMCs, ISG expression was associated with PLSCR1, TCF4, IRF9 and STAT1, prominent granulocyte infiltration was observed in kidneys of a murine lupus model (MRL/lpr mice), decreasing significantly after treatment with avacopan (a selective inhibitor of the C5a receptor).	Deng et al. (79)

*IFN, interferon; IL, interleukin; ISG, interferon inducible genes; LDG, low density granulocytes; LN, lupus nephritis; ND, not defined; PBMC, peripheral blood mononuclear cells; pDC, plasmacytoid dendritic cells; RNA-IC, RNA-immune complex; SLE, systemic lupus erythematosus*.

#### Peripheral Blood Immune Cells

A number of studies have already shown that the IFN type I cytokine family, involved in the immune response against viral infections, is importantly implicated in the pathogenesis of SLE. IFN I activates JAK/STAT signaling cascade leading to the induction of a variety of IFN-stimulated genes (ISGs), not only in the renal tissue of patients with LN, but also in leukocytes, obtained from peripheral blood of SLE patients (80, 81). Recently, a large scale study found that increased expression of ISGs in different immune cell subpopulations can distinguish pediatric SLE from healthy controls. Expansion of specific subpopulations enriched in ISGs was especially pronounced in pediatric, as well as adult SLE patients with the highest disease activity (74). In addition to type I IFNs, Hjorton et al. (75) demonstrated that RNA-containing immune complexes (RNA-ICs), found in patients with SLE, have the capacity to induce type III IFN (e.g., IFNλ1–3) production, which increased in the presence of GM-CSF, IL3, IL6 and IFNα2b, indicating that both type I and type III IFNs have a contributing role in SLE. Using scRNA-seq, they found that type III IFNs, dominated by IFNλ1, were exclusively expressed in a specific minor cluster of plasmacytoid dendritic cells (pDC), within a subset of the type I IFN expressing pDCs (75). The important role of IFNλ in immune dysregulation and tissue inflammation was later confirmed in a mouse model of TLR7-induced lupus. IFNλ receptor deletion resulted in significantly lower immune cell activation, and reduced skin and kidney damage of lupus mice. ScRNA-seq analysis of mice spleen and human peripheral blood revealed that only mice neutrophils and human B cells upregulate ISGs in response to stimulation with IFNλ. However, IFNλ was able to activate keratinocytes and mesangial cells to produce chemokines and promote inflammation in the skin and kidneys. IFNλ may thus exert its effects at barrier sites, rather than through peripheral immune cells (76).

Type I IFN signature is particularly enhanced in low density granulocytes (LDG), a subgroup of neutrophils, which have been shown to be implicated in the pathogenesis of SLE (82). In addition to upregulated IFN production, LDGs have increased neutrophil extracellular trap (NET) formation activity, pro-inflammatory effects and lower apoptosis rates compared to normal density granulocytes (83). Differential gene expression analysis revealed multiple upregulated ISGs in the LDG cluster compared with other cell clusters in SLE patients, indicating that LDGs significantly drive the type I IFN signature. There are two types of LDGs present in SLE patients: immature and intermediate mature LDGs with distinct transcriptional and epigenetic profiles, as well as functional properties. The presence of intermediate mature LDGs was associated with SLE organ damage, as well as the presence and severity of coronary artery disease (78). Analyzing several deposited scRNA-seq datasets, as well as their own experimental dataset, Deng et al. (79) confirmed that among SLE peripheral blood immune cells, LDGs exhibited the highest ISG activity, further pointing toward their prominent role in SLE pathogenesis. Their hypothesis that LDG and neutrophils infiltrate the kidneys during LN was confirmed in a lupus model of MRL/lpr mice. A prominent granulocyte infiltration was observed in the affected kidneys, decreasing significantly when mice were treated with avacopan (a selective inhibitor of the C5a receptor), which blocked C5a-mediated chemotaxis and granulocyte infiltration, as well as improved renal function (79).

### Systemic Sclerosis (SSc)

Systemic sclerosis is an incurable, chronic orphan disease characterized by inflammation, vascular changes, autoimmunity and fibrosis of the skin and internal organs, including the heart, kidneys and lungs (84). Interstitial lung disease (ILD) is a frequent complication of SSc (in up to 80% of patients), which can result in respiratory failure and death in about a third of patients (85, 86). The striking patient-to-patient variability and a lack of knowledge in understanding the mechanisms underlying inflammation and fibrosis in ILD are associated with very limited therapeutic options currently available for SSc-ILD patients and consequently with high mortality rates (87). Novel studies exploiting scRNA-seq data of SSc-ILD lung and skin tissues (Table 3) might provide a valuable insight into previously unknown subsets of cells that drive the pathogenesis of SSc and identify novel therapeutic targets.

**Table 3:** Application of scRNA-seq technology in systemic sclerosis.

**Sample type**	**Subjects or mouse model (number)**	**Cells sequenced (number)**	**Single cell platform**	**Main findings**	**Reference**
Human lung tissue	SSc-ILD patients (*n* = 4), healthy controls (*n* = 4)	Lung tissue cells (*n* = 56,196)	10X Genomics Chromium	Three fibroblast subpopulations: SPINT2^hi^, MFAP5^hi^, and WIF1^hi^, expanded myofibroblasts in SSc-ILD, with upregulated expression of collagens and other profibrotic genes.	Valenzi et al. (88)
Human and mice lung tissue	SSc-ILD patients (*n* = 2), IPF patients (*n* = 3), bleomycine induced SSc mice (*n* = 2), control mice (*n* = 2)	Human lung tissue cells (*n* = 83,316), murine lung tissue cells (*n* = 25,953)	10X Genomics Chromium	A specific cluster of fibroblasts present uniquely in murine and human fibrotic lungs with high expression of CTHRC1, localized within fibroblastic foci.	Tsukui et al. (89)
Human lung tissue	SSc-ILD patients (*n* = 8), IPF patients (*n* = 8), healthy controls (*n* = 5)	Lung tissue cells (*n* = 85,756)	10X Genomics Chromium	3 main subsets of macrophages: SPP1^hi^, FABP4^hi^ and FCN1^hi^. Type I IFN signaling was upregulated in SSc-ILD, IFNγ signaling was upregulated in IPF. AT1 exhibited the most distinct expression patterns between IPF and SSc-ILD, KRT5-/KRT17+ aberrant basaloid cells were identified in SSc-ILD.	Valenzi et al. (90)
Human lung tissue	SSc-ILD patients (*n* = 3)	FACS sorted CD45+, EPCAM+ and CD31+ lung tissue cells (*n* = 29,000)	10X Genomics Chromium	ICs activate monocytes to promote fibroblast migration through secretion of OPN, further amplified by MCSF and IL6. The levels of OPN are increased in the serum of SSc-ILD patients and its expression is significantly enriched in lung tissue macrophages.	Gao et al. (91)
Human skin tissue	SSc patients (*n* = 12), healthy controls (*n* = 10)	Skin tissue cells (*n* = 65,199)	10X Genomics Chromium	A new subcluster of fibroblasts in SSc, expressing PRSS23, SSc skin myofibroblasts co-expressed SFRP2 and SFRP4, SFRP2^hi^WIF1^−^ fibroblasts are the progenitors of myofibroblasts.	Tabib et al. (92)
Human skin tissue	SSc patient (*n* = 1), healthy control (*n* = 1)	Skin tissue cells (*n* = 184)	SmartSeq2	Endothelial cells in SSc were enriched in extracellular matrix generation, negative regulation of angiogenesis and EMT, the most upregulated genes in SSc were HSPG2, and APLNR.	Apostolidis et al. (93)
Human skin tissue	SSc patients (*n* = 12), healthy controls (*n* = 10)	Skin tissue cells (*n* = 65,199); myeloid cells (*n* = 2,465)	10X Genomics Chromium	Myeloid subpopulation in SSc skin that expressed monocyte markers (FCN1, EREG, S100A8 and S100A9) was associated with more severe skin disease. Proliferating macrophages and pDCs were determined almost uniquely in SSc skin.	Xue et al. (94)
Human skin tissue	SSc patients (*n* = 27), healthy controls (*n* = 10)	FACS sorted skin CD3+ T-cells (*n* = 3,729)	10X Genomics Chromium	Identified a cluster of recirculating CXCL13^+^ T cells uniquely detected in SSc skin, with gene expression profile similar to Tfh cells, adjacent to CD20^+^ B cells within inflammatory infiltrates in the skin, lower frequency of CD3^+^CXCL13^+^ cells in SSc patients treated with immunosuppressive drugs.	Gaydosik et al. (95)

*AT1, alveolar type 1; EMT, epithelial-to-mesenchymal transition; FACS, flow-activated cell sorting: IC, immune complex; IFN, interferon; IL, interleukin; ILD, interstitial lung disease; IPF, idiopathic pulmonary fibrosis; MCSF, macrophage colony-stimulating factor; OPN, osteopontin; pDC, plasmacytoid dendritic cells; SSc, systemic sclerosis*.

#### Lung Tissue-Derived Cells

In 2019, Valenzi et al. (88) analyzed lung tissue-derived cells from SSc-ILD patients and healthy controls, focusing on fibroblasts and myofibroblasts, which play a key role in fibrosis due to their capacity of extracellular matrix depositing and remodeling. A great expansion of myofibroblasts (expressing high levels of ACTA2) appeared in SSc-ILD samples, including a subpopulation of actively proliferating myofibroblasts. Myofibroblasts phenotypically changed in SSc-ILD and significantly upregulated expression of collagens and other profibrotic genes. While myofibroblasts can derive from multiple sources in pathological states, it was anticipated that MFAP5^hi^ fibroblasts may act as their progenitors in SSc-ILD (88). In 2020, Tsukui et al. (89) utilized scRNA-seq to characterize all collagen-producing cells in normal and SSc-ILD lungs. Using a murine model with bleomycin-induced SSc, they identified a specific cluster of fibroblasts, mostly found in fibrotic murine lungs. This cluster expressed collagen triple helix repeat containing protein 1 (CTHRC1), previously shown to be increased in the lungs of patients with fibrosis. ScRNA-seq of human normal and fibrotic lungs revealed that CTHRC1-expressing fibroblasts were uniquely present in fibrotic lungs and localized within fibroblastic foci. This fibroblast subpopulation exhibited enhanced migratory properties and could be importantly implicated in the development of fibrosis in mice and humans (89).

Idiopathic pulmonary fibrosis (IPF) is another form of ILD of unknown etiology, sharing some similar aspects and mechanisms of development with SSc-related ILD. The significant role of inflammation is more established in the pathogenesis of SSc-ILD, while investigating the shared and distinct mechanisms between SSc-ILD and IPF, may yield important new insights influencing therapeutic development for both diseases (96). Sequencing single cells from IPF, SSc-ILD and healthy lung tissue, Valenzi et al. (90) identified three main subsets of macrophages: SPP1^hi^, FABP4^hi^, and FCN1^hi^ monocyte-derived macrophages. Type I IFN signaling together with increased expression of ISGs were significantly upregulated in macrophages obtained from SSc-ILD patients compared to IPF, while IFNγ signaling was upregulated in macrophages, cytotoxic T cells, and natural killer cells of IPF. Alveolar type 1 (AT1) cells were decreased in both diseases compared to healthy tissue and exhibited the most distinct expression patterns between IPF and SSc-ILD. SSc-ILD lung tissue-derived cells showed deregulated expression of genes involved in protein ubiquitination and catabolism, as well as cellular response to oxygen levels, suggesting that cell stress leads to death of AT1 cells. The authors identified for the first time an aberrant subset of KRT5^−^KRT17^+^ basaloid cells with high expression of markers of cellular senescence and epithelial mesenchymal transition in SSc-ILD. Compared to IPF, SSc-ILD patients had upregulated genes involved in vasculogenesis, prostaglandin biosynthesis, and platelet-derived growth factor receptor signaling, implicating a significant expansion of the endothelium (90).

#### Immune Complexes

Various studies demonstrated the presence of immune complexes (ICs) in the sera, lungs, and bronchoalveolar lavage (BAL) fluid of SSc patients, indicating their potential role in disease pathogenesis, possibly through myeloid cell activation (97–99). Gao et al. (91) confirmed this hypothesis, showing evidence that ICs activate monocytes to promote lung fibroblast migration through secretion of osteopontin (OPN), which was potentiated by monocyte colony stimulating factor (MCSF) and IL6. The levels of OPN were increased in the serum of SSc-ILD patients and its expression was significantly enriched in lung tissue macrophages, as demonstrated by scRNA-seq. OPN has a potential to be used as a systemic biomarker to predict future SSc-ILD progression, as well as a novel therapeutic target (91).

#### Skin Tissue-Derived Cells

The majority of SSc patients develop skin fibrosis in earlier stages of the disease. As in lung fibrosis, the development of skin fibrosis is also driven mainly by fibroblasts and myofibroblasts (100). A largescale study by Tabib et al. (92) showed that the transcriptional profile of SSc dermal fibroblasts changed significantly compared to healthy dermal fibroblasts. They identified a new fibroblast subcluster from SSc patients that expressed PRSS23 and had upregulated expression of genes involved in extracellular matrix and collagen fibril organization, wound healing, and skeletal system development. Within the PRSS23^+^ cluster, a second population (SFRP2^+^, SFRP4^+^), identified as myofibroblasts, was found exclusively in SSc skin. The pseudo-time analysis showed that the SFRP2^hi^ fibroblasts were the immediate progenitors of myofibroblasts, however only a fraction of SFRP2^hi^ SSc fibroblasts differentiated into myofibroblasts, which is driven by upstream transcription factors, including FOSL2, RUNX1, STAT1, FOXP1, IRF7, CREB3L1, and SMAD3 (92). A subsequent study by the same group focused on skin myeloid cell populations and revealed 12 myeloid cell clusters, three of which were specifically identified in SSc skin. One SSc-associated cluster consisted of macrophages that expressed high levels of FCGR3A, while the second SSc-associated myeloid cluster expressed various monocyte markers. The presence of the latter cluster was was associated with more severe skin disease. Proliferating macrophages and dendritic cells were determined almost uniquely in SSc skin. Gene expression profiles in these and other myeloid subclusters revealed high expression of chemokines and enrichment in processes associated with innate immune activation, possibly through toll-like receptors (TLRs). However, there was significant variability in the appearance and activation status of myeloid cells observed between patients, suggesting different underlying mechanisms of pathogenesis and/or temporal disease activity (94).

Some of the most evident clinical characteristics of SSc, including Raynaud's phenomenon, telangiectasias, and pulmonary arterial hypertension, develop as a consequence of vascular injury and the underlying endothelial dysfunction (101, 102). The endothelial cell gene expression profile in the skin of SSc patients was found to be enriched in processes associated with extracellular matrix generation, negative regulation of angiogenesis and epithelial-to-mesenchymal transition. Among the most upregulated genes in SSc skin vs healthy controls were HSPG2, an extracellular matrix protein, induced by TGFβ, and APLNR, that plays a role in angiogenesis. These two proteins have not been previously associated with SSc pathogenesis, however, they have been linked to vascular dysfunction and fibrosis in different settings, making them of interest in further SSc studies (93).

Several T-lymphocyte subsets and associated cytokines have been implicated in the inflammatory and fibrotic processes of SSc, however their heterogeneity in SSc skin has only recently been revealed by Gaydosik et al. (95). They detected several subsets of infiltrating, as well as tissue-resident T-lymphocytes in healthy and SSc skin that were enriched in different signaling pathways. A cluster of recirculating CXCL13^+^ T cells was uniquely detected in SSc skin. These cells had a gene expression profile similar to that of Tfh cells, although they lack the canonical Tfh expression of CXCR5 and BCL6. They were found adjacent to CD20^+^ B cells within inflammatory infiltrates in the skin, indicating they might promote B cell responses. A significantly lower frequency of CD3^+^CXCL13^+^ cells was detected in SSc patients treated with immunosuppressive drugs, compared with untreated patients. This indicates that a more targeted T cell-based therapy might be used in SSc patients, resulting in an improved efficacy and lower toxicity (95).

### Other Rheumatic Diseases

#### Psoriatic Arthritis

Psoriatic arthritis (PsA) is an inflammatory arthritis of the joints, affecting approximately one third of patients suffering from skin psoriasis. The pathogenesis of PsA is complex and involves multiple cell types, such as osteoblasts and osteoclasts, as well as immune cells within the joint synovial lining tissue and/or fluid (103). ScRNA-seq of synovial fluid from affected joints of PsA patients revealed that the predominant cell types identified belong to a monocyte/macrophage cluster, representing classical, non-classical and intermediate cells. Compared to RA and OA, the frequency of CD14^+^CD6^−^ classical monocytes/macrophages was reduced, while the frequency of CD14^+^CD16^+^ intermediate monocytes/macrophages was increased in the synovial fluid of PsA patients (Table 4). Protease-activated receptor 2 (PAR2) expression was found within monocytes/macrophage clusters and consistent with this, serine proteinases that bind to PAR2, were detected in PsA synovial fluid. Monocyte/macrophage PAR2 activation by tryptase-6 resulted in secretion of high levels of monocyte chemoattractant protein-1 (MCP-1). The invading macrophages can thus both produce and respond to tryptase-6 via PAR activation, and mediate further recruitment of peripheral monocytes/macrophages into the inflamed joint, leading to sustained inflammation and disease progression. Typtase-6-PAR2 signaling pathway may thus represent a novel therapeutic target in PsA (104).

**Table 4:** Application of scRNA-seq technology in PsA, AxSpA, SjS and KD.

**Sample type**	**Subjects or mouse model (number)**	**Cells sequenced (number)**	**Single cell platform**	**Main findings**	**Reference**
Human synovial fluid	PsA patients (*n* = 3)	Synovial fluid cells (ND)	10X Genomics Chromium	Reduced CD14+CD6- classical and increased CD14+CD16+ intermediate monocytes/macrophages in PsA, Monocyte/macrophage PAR2 activation by tryptase-6 resulted in increased secretion of MCP-1.	Abji et al. (104)
Human synovial tissue, synovial fluid and peripheral blood	PsA patients (*n* = 9)	FACS sorted CD4^+^CD8^+^ T cells (*n* = 41,202 from PBMC and synovial fluid; *n* = 251 from synovial tissue)	10X Genomics Chromium, SmartSeq2	16 clusters of memory CD4 and CD8 T cells in synovial fluid, a specific cluster of synovial CD8 T cells with higher expression of MKI67 and STMN111. T-cell receptor alpha-chain gene TRAV27 was significantly upregulated in the CD8 T cell cluster. The expanded CD8 T cell population was characterized by increased expression of CXCR3, while CXCL9 and CXCL10, were elevated in PsA synovial fluid.	Penkava et al. (105)
Human peripheral blood	AxSpA (*n* = 2), CD (n = 2), CD-axSpA (*n* = 2) patients, healthy controls (*n* = 2)	FACS sorted CD45^+^ leukocytes (*n* = 50,580)	10X Genomics Chromium	CD-axSpA patients showed an expansion of mature GZMB+ T cells f both CD4+ and CD8+ lineages in the peripheral blood, a prominent IFN activation signature and elevated plasma levels of IFN-y and IL-6.	Lefferts et al. (106)
Human peripheral blood	SjS patients (*n* = 5), healthy controls (*n* = 5)	Isolated PBMC (*n* = 57,288)	10X Genomics Chromium	Significant expansion of CD4+ cytotoxic T-lymphocytes and CD4+ TRAV13-2+ T cell in SjS, upregulated type I and II IFN signaling, and increased expression of ISGs (IFITM3, IFITM2, IFITM1, and XAF1).	Hong et al. (107)
Human peripheral blood	KD child (*n* = 1), healthy child (*n* = 1)	Isolated PBMC (*n* = 19,197)	10X Genomics Chromium	Identified 14 cell clusters in KD samples, expanded populations of NKT cells and plasmacytoid dendritic cell, lower frequency of naïve CD8+ T cells, T helper cell, B cells, multilymphoid progenitor cells in KD child. Major limitations: small samples size and lack of a validation cohort.	Fan et al. (108)
Human peripheral blood	KD children (*n* = 2), healthy children (*n* = 2)	Enriched monocytes (*n* = 8,880)	BD Rhapsody	CD14^+^CD16^−^ classical, CD14^+^CD16^+^ intermediate and CD14^−^CD16^+^ nonclassical monocytes were found in KD, classical monocytes were significantly expanded and expressed higher levels of SELL and MALAT, and lower levels of CXCL8 and JUN, classical monocytes in KD are less differentiated compared to their counterparts in healthy children.	Geng et al. (109)

*AxSpa, axial spondyloarthritis; CD, Chron's disease; FACS, flow-activated cell sorting; GZMB, granzyme B; IFN, interferon; IL, interleukin; KD, Kawasaki disease; ND, not defined; NKT, natural killer T cells; PBMC, peripheral blood mononuclear cells; PsA, psoriatic arthritis; SjS, Sjogren's syndrome*.

A large scale study used complementary single cell approaches to study leukocytes from the affected joints and peripheral blood of PsA patients. They reported on 16 clusters of memory CD4^+^ and CD8^+^ T cells in synovial fluid of affected joints. A specific cluster of synovial CD8^+^ T cells exhibited higher expression of the proliferation markers, indicating their active proliferation within inflamed joints. Increased expression of the MHCII genes HLA-DRB1 and HLA-DRA, as well as the effector molecules granzyme A (GZMA) and granzyme B (GZMB) was observed in synovial fluid-derived CD8^+^ T cells, as compared to peripheral blood-derived CD8^+^ T cells. Furthermore, the T cell receptor alpha chain gene TRAV27 was also significantly upregulated in the synovial fluid CD8^+^ T cell cluster, indicating a clonal expansion of this subpopulation in the synovium that was confirmed by scRNA-seq. The expanded CD8^+^ T cell population was characterized by increased expression of chemokine receptor CXCR3, while two CXCR3 ligands, CXCL9 and CXCL10, were elevated in PsA synovial fluid (105).

#### Axial Spondyloarthritis

Axial spondyloarthritis (axSpA) is an inflammatory disease of the axial skeleton associated with significant pain and disability. AxSpA frequently occurs together with inflammatory bowel diseases (IBD) and the overlapping characteristics present significant challenges in the diagnosis and treatment approaches in individuals afflicted simultaneously with both diseases (110). Performing scRNA-seq in PBMCs from patients with axSpA, Crohn's disease (CD) and a combination of both, Lefferts et al. (106) discovered an expansion of mature GZMB^+^ T cells of both CD4^+^ and CD8^+^ lineages in the peripheral blood of CD-axSpA patients compared to patients with either CD or axSpA (Table 4). Furthermore, a prominent IFN signature was observed in all T cell populations from CD-axSpA patients, together with elevated plasma levels of IFNγ and IL6. These data demonstrated that fundamental immunological differences exist between CD-axSpA, axSpA and CD, indicating that CD-axSpA is a distinct disease entity, requiring distinct therapeutic approaches for effective treatment (106) (Table 4).

#### Sjögren's Syndrome

Patients with Sjögren's syndrome (SjS), an autoimmune disease of the exocrine glands, are characterized with oral and ocular dryness, resulting from extensive lymphocytic infiltration of the salivary and lacrimal glands (111). Approximately 30–40% of patients develop systemic manifestations that involve the kidneys, lungs, and nervous system (112). The disease is very heterogeneous, which limits the complete understanding of the pathogenic mechanisms, and complicates the discovery of novel therapeutic targets. Furthermore, in certain cases, it may progress to non-Hodgkin's lymphoma. Hong et al. (107) identified two CD4^+^ T cell subpopulations that were significantly expanded in peripheral blood of patients with SjS (Table 4). The first subpopulation was characterized by high expression of cytotoxicity genes (CD4^+^ cytotoxic T-lymphocytes), and the second had increased expression of T cell receptor (TCR) variable genes (CD4^+^ TRAV13-2^+^ T cells). Upregulated type I and II IFN signaling, together with increased expression of ISGs was found in most immune cells in SjS patients. The specific expansion of CD4^+^ cytotoxic T-lymphocytes may be important for disease development and its depletion might be a promising treatment strategy (107).

#### Kawasaki Disease

Kawasaki disease (KD) is an acute systemic vasculitis, predominantly affecting vessel walls of medium-sized arteries and typically occurs in children. The disease is more frequent in children of Asian origin and is the most common cause of acquired heart disease in children in developed countries (113). Although the etiology of KD remains unknown, several studies have shown that it might develop as a consequence of an aberrant immune response (114). This was confirmed by two different studies performing scRNA-seq in PBMCs of KD patients (108, 109) (Table 4). The first one identified 14 cell clusters in KD samples, with expanded populations of natural killer T (NKT) cells and pDC, and lower frequency of naïve CD8^+^ T cells, T helper cells, B cells, and lymphoid progenitor cells. Although the study provided evidence of immune dysregulation in KD patients, there is a significant limitation of including only one KD patient and one healthy control, as well as the lack of a larger validation cohort (108). The second study focused on monocyte subsets, and discovered three monocyte subpopulations in KD children, including CD14^+^CD16^−^ classical, CD14^+^CD16^+^ intermediate and CD14^−^CD16^+^ nonclassical monocytes. In KD patients, classical monocytes were significantly expanded compared to healthy children. Trajectory analysis showed that classical monocytes in KD eventually differentiated into nonclassical monocytes. This transition occurred through intermediate monocytes, as well as through a phenotype more similar to classical monocytes found in healthy children. Classical monocytes in KD might thus have a less differentiated phenotype compared to their healthy counterparts. Altered monocyte subpopulations might be considered as biomarkers for KD diagnosis and treatment (109).

### A Step Toward Precision Medicine in Rheumatic Diseases

An important unmet need in rheumatic autoimmune inflammatory diseases is the identification of cell and/or molecular biomarkers that would predict which drug would be most appropriate for an individual or a group of patients to achieve disease remission sooner. The frequently utilized trial and error, “one- size- fits-all” therapeutic approach often leads to drug failure and is associated with reduced patients' quality of life, as well as presents a substantial financial burden. Hence, it should be replaced with a more targeted approach, that could ensure the best possible management of the disease for individual patients (115), which is also the ultimate goal of precision medicine. A prerequisite for successful implementation of precision medicine into the clinical setting is identification of biomarkers, that would stratify patients into responder/nonresponder groups. The clinical utility of scRNA-seq for the purposes of precision medicine have already been demonstrated in many biomedical fields, especially in oncology, and such studies provide valuable and helpful information, especially regarding experimental design and data analysis (38, 116). Notably, existing scRNA-seq studies, described in this review, have already enabled discoveries of novel subsets of cells with distinct anatomical positions, transcriptional profile and function in humans, as well as in mouse models of rheumatic diseases (Tables 1–4). Moreover, enrichment of certain cell types with specific transcriptional profiles in the affected tissue (e.g., specific clusters of synovial macrophages) has been associated with disease remission or relapse after using a conventional therapeutic approach in RA (60), while ISG-enriched subpopulations of PBMCs were associated with high disease activity in SLE patients (74). Although this abundance of data already revealed some promising biomarkers, additional studies with molecular characterization of the disease on both systemic and local, tissue levels are required. Furthermore, tissue-based *in vitro* models, coupled with scRNA-seq will enable better understanding of the complex mechanism of drug failure or response, as well as help identify novel therapeutic targets. For example, *ex vivo* tissue models, 3D models, or organoids could be used to identify novel drug candidates or serve as screening platforms (117). A 3D organoid co-culture of synovial fibroblasts and endothelial cells has yielded results (58) of the NOTCH3 signaling cascade, that could be an important novel therapeutic target in RA. Another possibility is to use patient-derived cells *ex vivo* (i.e., in organoids) to predict individual patient drug responses (118). This was demonstrated by Kuo et al. (59) who used dissociated synovial tissue-derived cells from RA patients, for testing currently used anti-inflammatory therapies. The response of synovial macrophages to each drug resulted in a distinct gene expression pattern, which may ultimately affect the patient's outcome. This type of patient-oriented and molecular-driven approach could inform and guide the design and patient selection for future more narrowly tailored clinical trials. To achieve the ultimate goal of controlling the disease activity more rapidly and reduce the economic, as well as personal burden of rheumatic diseases, innovative patient-centric, molecular pathology-driven clinical trial approaches are needed (119).

## Conclusion

ScRNA-seq has enabled us to analyse in depth the complex cellular and molecular networks at the resolution of an individual cell in the heterogeneous tissue microenvironment. Additionally, computational tools analyzing scRNA-seq data offered us the opportunity to predict trajectories of different cell states, transitions and differentiation in the tissue during physiological and pathological processes. With the possibility to obtain massive amounts of data from individual cells, scRNA-seq has exceeded other traditional methods, such as flow cytometry, bulk RNA-seq and immunohistochemistry. The technique is particularly important for studying diseases with high patient heterogeneity, including autoimmune rheumatic disorders. However, the translation of novel findings from single cell approaches into advanced diagnostic tools and treatments in rheumatic and other diseases has yet to be realized. There are still several challenges to overcome in order to provide valuable information for support of clinical decision-making. One challenge is the complexity of high dimensional single cell multiomics data which, for a successful and meaningful clinical interpretation, requires the interdisciplinary collaboration of bioinformatics, computational scientists, biologists and clinicians. Another challenge is the high cost of reagents and equipment, limiting the ability to profile large cohorts of patients with different clinical presentations and demographic backgrounds, as well as the integration of scRNA-seq and other single cell omics and high throughput data into a user-friendly interface that is easily accessible and available to the entire research community. To overcome these challenges, multicenter collaborations, such as in ELIXIR (https://elixir-europe.org/) an intergovernmental organization that brings together life science infrastructural resources from across Europe, are needed, coupled with practical and standardized protocols for wet-lab personnel, as well as bioinformatic pipelines.

Despite several challenges, which will likely be resolved with further development of the technology, as well as with tighter collaborations between wet-lab, dry-lab and clinical experts, the translational potential of scRNA-seq in rheumatic inflammatory diseases is obvious. Better understanding of the composition and functional states of tissue resident stromal and immune cells, together with their complex networks and interactions will provide further insights into disease development and progression, determine underlying mechanisms of drug resistance, as well as identify candidate therapeutic targets with low rates of off-target effects. With the recent availability of spatial transcriptomics, it will now be possible to explore gene expression signatures within the positional context of those cells in a tissue (120, 121). Furthermore, cellular indexing of transcriptomes and epitopes by sequencing (CITE-seq), a method initially utilized for the analyses of cord blood and PBMCs, could easily be adapted to study the immune systems in patients with rheumatic diseases. Here, oligonucleotide-labeled antibodies that contain poly(A) tails are used to simultaneously determine cell surface proteins and transcriptomes. Several commercially available DNA-barcoded antibodies and fully validated sample processing platforms are already available for this purpose (51, 122). The application of several single cell omics, spatial transcriptomics and data integration from patients, animal and experimental models is likely to optimize the path toward precision medicine in autoimmune and inflammatory rheumatic diseases in the near future.

## Author Contributions

TK wrote the manuscript. SS-Š, BL, and PF contributed to specific parts of the review and revised and approved the final version of the manuscript. All authors contributed to the article and approved the submitted version.

## Funding

The research for this paper was supported by Slovenian Research Agency (Grant Number #P3-0154).

## Conflict of Interest

The authors declare that the research was conducted in the absence of any commercial or financial relationships that could be construed as a potential conflict of interest.

## Publisher's Note

All claims expressed in this article are solely those of the authors and do not necessarily represent those of their affiliated organizations, or those of the publisher, the editors and the reviewers. Any product that may be evaluated in this article, or claim that may be made by its manufacturer, is not guaranteed or endorsed by the publisher.
